# Do people with diabetes have a higher risk of developing postoperative endophthalmitis after cataract surgery? A systematic review and meta-analysis

**DOI:** 10.1186/s12348-025-00483-9

**Published:** 2025-03-12

**Authors:** Kai-Yang Chen, Hoi-Chun Chan, Chi-Ming Chan

**Affiliations:** 1 School of Medicine, College of Medicine, Taipei Medical University, Taipei, Taiwan; 2 School of Pharmacy, China Medical University, Taichung, Taiwan; 3 Department of Ophthalmology, Cardinal Tien Hospital, New Taipei City, Taiwan; 4 School of Medicine, Fu Jen Catholic University, New Taipei City, Taiwan

**Keywords:** Postoperative endophthalmitis, Diabetes mellitus, Cataract surgery, Posterior capsule rupture, Antibiotic prophylactic, Meta-analysis

## Abstract

**Purpose:**

Postoperative endophthalmitis (POE) is a rare but severe complication of cataract surgery. While diabetes mellitus may increase the risk of POE, the relationship remains unclear.

**Methods:**

A systematic review and meta-analysis were conducted following PRISMA guidelines. PubMed, Scopus, Medline, Embase, and Google Scholar were searched for relevant studies up to September 10, 2024. The study included both randomized controlled trials and observational studies that evaluated POE outcomes in cataract surgery patients, comparing people with and without diabetes. Random-effects models were used to calculate pooled odds ratios (OR) with 95% confidence intervals (CI).

**Results:**

Nine studies were included in the systematic review, with seven analyzed in the meta-analysis. The review on POE in people with diabetes undergoing cataract surgery revealed a higher incidence in this group, with a pooled odds ratio (OR) of 1.174 (95% CI: 1.109 to 1.242; p = 0.000) and an incidence rate of 0.261%, compared to 0.242% in people without diabetes. Males with diabetes had a 1.634 times higher risk of POE (p = 0.048), while diabetes and hypertension together increased risk by 3.961 times (p < 0.001). Posterior capsule rupture (PCR) was associated with a significantly higher risk of developing POE, which was also more common in people with diabetes, with an OR of 3.434 (95% CI: 1.789 to 6.591; p = 0.0001). The use of postoperative intracameral and topical antibiotics significantly reduced the risk of POE in both people with and without diabetes (OR: 0.231; p = 0.00).

**Conclusions:**

This meta-analysis shows that people with diabetes undergoing cataract surgery have a significantly higher risk of POE and PCR compared to those without diabetes, with odds ratios of 1.174 and 3.434, respectively. The administration of intracameral and topical antibiotics significantly reduces the risk of POE in both groups. Our study highlights the importance of maintaining well-controlled blood sugar and blood pressure before surgery. Additionally, extra caution should be taken during surgery to prevent PCR, and appropriate antibiotic use should be considered to minimize the risk of POE.

## Introduction

Cataract surgery is one of the most frequently performed surgical procedures worldwide, significantly improving visual acuity and quality of life [1–3]. However, it carries certain risks, with postoperative endophthalmitis (POE) being one of the most serious complications [4, 5]. This rare but vision-threatening infection can lead to severe visual impairment or even blindness. The incidence of POE ranges from 0.02% to 0.26%, influenced by patient-specific and procedural factors [6]. Diabetes mellitus is a recognized risk factor for POE due to its association with delayed wound healing, heightened inflammatory responses, and compromised immunity [7]. People with diabetes often have coexisting conditions such as hypertension and systemic complications like diabetic retinopathy, further increasing their susceptibility to post-surgical complications [8]. Poor blood sugar control has been linked to a higher risk of surgical site infections across various procedures [9]. Additionally, insulin use, particularly in advanced diabetes, has been associated with an increased infection risk, possibly due to impaired immune function and delayed healing [10].

Despite these concerns, the relationship between diabetes, blood sugar control, and POE following cataract surgery remains unclear. As the number of people with diabetes undergoing cataract surgery rises due to the increasing prevalence of both conditions in aging populations, understanding the association between diabetes and POE is essential [11]. However, existing studies on POE incidence in this population have produced mixed results [12].

Elevated blood sugar levels and posterior capsule rupture (PCR) are potential contributors to POE risk [13, 14]. PCR, which occurs when the posterior lens capsule breaks during surgery, compromises the eye’s protective barrier, increasing the likelihood of infection [15]. This rupture can cause inflammation and vitreous prolapse, exposing the vitreous cavity to external contaminants, leading to POE [16, 17]. Additionally, the increased surgical complexity and prolonged exposure associated with PCR further elevate infection risk [18]. The study by Low et al. (2023) [19] found that patients who experienced PCR during cataract surgery had a significantly higher risk of developing POE, with an odds ratio of 7.111, meaning they were over seven times more likely to develop POE than those without PCR. This strong association underscores PCR as a critical risk factor that cataract surgeons should consider in both preoperative planning and intraoperative management to minimize POE risks.

While antibiotics are commonly used to prevent POE, it remains unclear whether their protective effects differ between people with and without diabetes [20]. Given the increasing frequency of cataract surgeries in this population, a thorough evaluation of risk factors and outcomes is crucial to improving perioperative care and reducing complications [21].

This study aims to enhance our understanding of how diabetes affects the likelihood of POE after cataract surgery. Given the conflicting findings in existing research and the increasing number of people with diabetes undergoing cataract surgery, this study addresses the knowledge gap by examining POE risk and related factors in this population compared to people without diabetes. The primary objective is to determine the incidence rate of POE in people with and without diabetes who undergo cataract surgery. Additionally, this study explores the impact of PCR and antibiotic use on POE risk, with the ultimate goal of improving perioperative care strategies for people with diabetes.

## Methods

### Study design and selection criteria

This systematic review and meta-analysis followed the Preferred Reporting Items for Systematic reviews and Meta-Analyses (PRISMA) guidelines [22]. A comprehensive literature search was conducted across multiple databases, including PubMed, Scopus, Medline, Embase, and Google Scholar, up to September 5, 2024, to identify studies evaluating POE incidence and risk factors in people with and without diabetes undergoing cataract surgery. Eligible studies included randomized controlled trials (RCTs), case–control studies, cohort studies, and case series published in peer-reviewed journals. Studies were being included if they reported POE incidence, risk factors, or relevant surgical outcomes. The study protocol was registered in PROSPERO (CRD42024556737).

### Search strategy

A systematic search strategy was employed, utilizing keywords and Medical Subject Headings (MeSH) terms such as "postoperative endophthalmitis," "diabetes," "cataract surgery," "risk factors," and "incidence." The search was limited to articles published in English, with no restrictions on publication date. Duplicates were removed and titles and abstracts were screened to identify potentially eligible studies. Full-text articles were reviewed to confirm eligibility based on the predefined inclusion criteria.

### Inclusion criteria

This analysis focuses on studies published in peer-reviewed journals, including randomized controlled trials (RCTs), case–control studies, cohort studies, and case series, that investigate POE incidence and risk factors in people undergoing cataract surgery. The study population (P) includes both people with and without diabetes who undergo cataract surgery. The intervention (I) examines antibiotic use as a potential factor influencing POE risk. The comparison (C) evaluates POE incidence between the two groups. The outcome (O) centers on research reporting POE incidence and associated risk factors. The primary exposure analyzed is antibiotic use as a risk factor for POE. This analysis directly compares POE incidence in people with and without diabetes while assessing related risk factors.

### Data extraction and quality assessment

Data were extracted from the selected studies using a standardized form, which included information on the study design, sample size, patient demographics, risk factors, and outcomes related to POE. Two independent reviewers (K.Y.C and H.C.C.) conducted data extraction to minimize bias. Discrepancies were resolved by discussion or consultation with a third reviewer (C.M.C). The methodological quality of the included studies was assessed using appropriate tools based on the study design: Cochrane Risk of Bias Tool for RCTs and ROBINS-I for observational studies.

### Statistical analysis

Statistical analyses were conducted using the Comprehensive Meta-Analysis software (version 3.7). A random-effects model was used to account for the variability among the studies. The odds ratio (OR) with a 95% confidence interval (CI) was calculated for each outcome, with p < 0.05 considered statistically significant. Heterogeneity among the studies was assessed using the I^2^ statistic, with a threshold of greater than 50% indicating significant heterogeneity. Publication bias was evaluated using funnel plots and Egger’s test to determine the symmetry of data distribution.

## Results

### Literature search

**Fig. 1:**
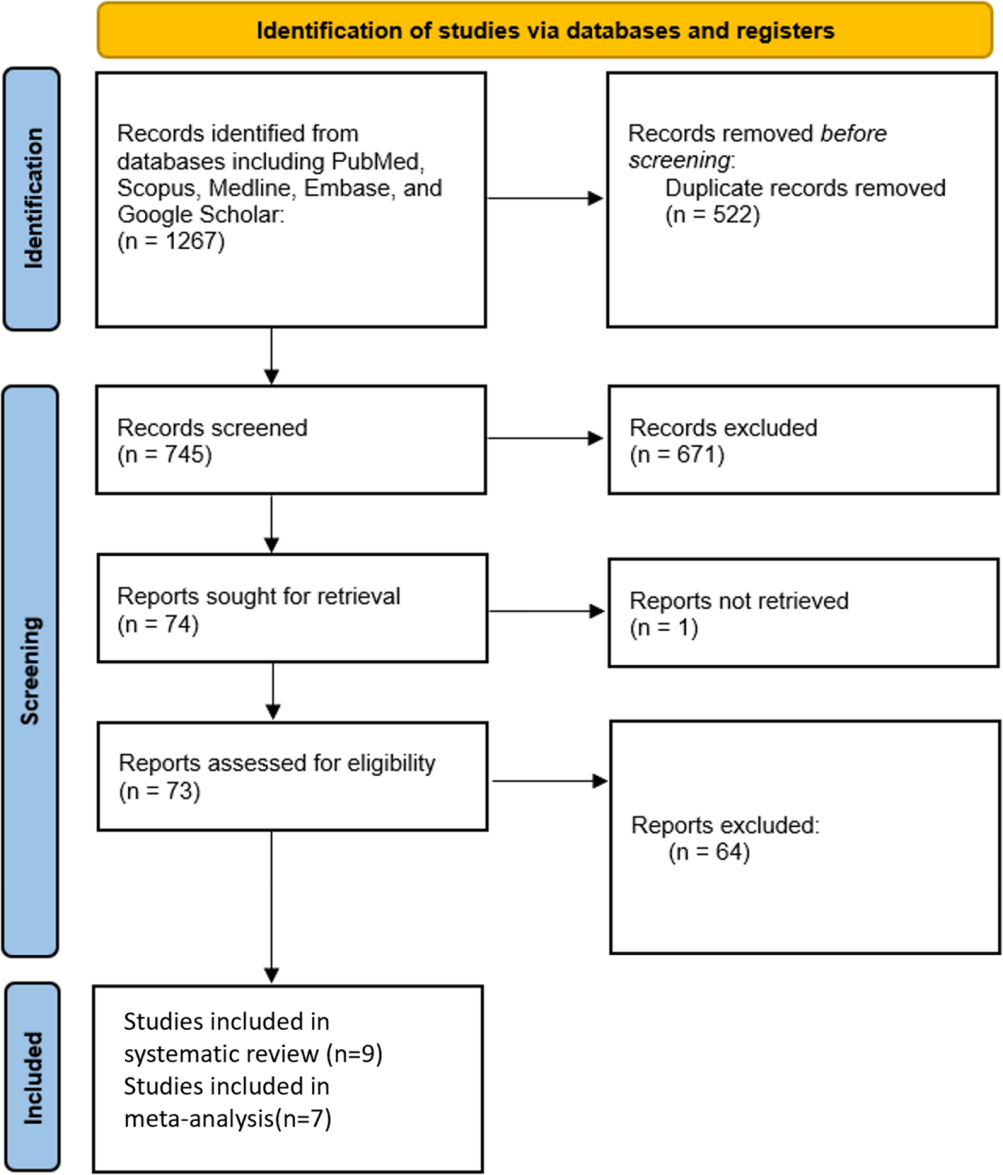
PRISMA flow diagram illustrating the study selection process for systematic review and meta-analysis

A comprehensive literature search identified 1267 records from multiple databases including PubMed, Scopus, Medline, Embase, and Google Scholar. After the removal of duplicates and initial exclusions based on eligibility criteria, 745 records remained for further screening. Of these, 74 studies underwent a detailed eligibility assessment, while others were excluded due to irrelevant outcomes or a lack of sufficient detail. Ultimately, nine [23–31] studies were suitable for inclusion in this systematic review, with seven [23–27] incorporated into the meta-analysis (Fig. 1).

### Baseline characteristics

The baseline characteristics of the included studies, as shown in Table 1, demonstrated significant variability in the study design, location, and patient demographics. The sample sizes ranged from 11 to 1,263,497, with studies conducted across the USA, India, China, the UK, and Taiwan. The mean or median age of the participants was generally in the range of 54–73 years, and gender distribution varied. Insulin therapy was specifically reported by Silpa-Archa et al. (2019) [28], in which 23 (12%) out of 194 people with diabetes were on insulin, although other studies did not consistently report this detail. The most common causative organisms across studies included Staphylococcus epidermidis, Staphylococcus aureus, and Streptococcus species, although several studies, such as Sun et al. (2021) [24] and Senthamizh et al. (2022) [30], have noted a high prevalence of negative cultures. Despite differences in methodology and settings, these studies focused on cataract surgeries and postoperative infections, especially in people with diabetes.

**Table 1:** Baseline characteristics of included studies

Study name	Study Design	Country	Sample Size	Population	Gender (Female)	Age	Insulin Therapy	Causative Organism	Surgery	Outcome	Key Findings	Conclusion
Silpa-archa et al., 2019 [28]	Observational	USA	15/179	People undergoing cataract surgery	125	65 (36–97)	23	Staphylococcus epidermidis (5), Staphylococcus aureus (1), Streptococcus viridans (1), Enterococcus faecalis (1), Escherichia coli (1)	Cataract surgeries	Risk factor for postoperative endophthalmitis (PCE) identified as insulin treatment	Pre-operative glycaemic markers (HbA1c and fasting glucose) should be studied to confirm their role in PCE	Insulin treatment was the only identified risk factor for endophthalmitis. More studies on glycaemic markers needed
Gondhale et al., 2021 [23]	Observational	India	391	People undergoing cataract surgery	160	54.7	NR	Staphylococcus epidermidis (31), Viridans streptococci (4), Pseudomonas aeruginosa (6), No growth(73)	Cataract surgeries	POE incidence and factors	Men with diabetes had 1.634 times higher odds of POE, diabetes + hypertension increased odds by 3.961	Diabetes and hypertension, as well as rapid blood sugar reduction, increase the risk of postoperative endophthalmitis
Sun et al., 2021 [24]	Observational	China	55,612	People undergoing cataract surgery	32,933	≤ 44 (3/1561), 45–59 (8/7891), 60–74 (24/28,013), ≥ 75 (7/18,147)	NR	Negative (17), Coagulase-negative Staphylococci (1), Staphylococcus aureus (2), Enterococci (1), Pseudomonas species (1), Streptococcus (2)	Cataract surgeries	POE incidence over 12 years	Posterior capsule rupture, absence of antibiotics, and no lens washout increase POE risk	The incidence of endophthalmitis decreased over time due to better surgical practices
Dev et al., 1999 [29]	Observational	USA	11	People with DM	3	68	NR	NR	Vitrectomy, vitreous biopsy	Risk of retinopathy progression after endophthalmitis	Diabetic retinopathy worsened after endophthalmitis; poorer visual outcomes in these people	Inflammation may exacerbate diabetic retinopathy after endophthalmitis, leading to worse visual outcomes
Doft et al., 2001 [25]	Randomized controlled trial	USA	420	Endophthalmitis suspected people following a cataract surgery	241	73 (24–97)	NR	Gram Negative (2), Gram-positive (13), No growth (9), Gram-positive coagulase negative (34)	Cataract surgeries	POE treatment outcomes	Tap/biopsy or vitrectomy as treatment options for people with diabetes better than LP vision	More trials are needed to determine the best treatment option for people with diabetes better vision
Kamalarajah et al., 2007 [26]	Observational	UK	214	Individuals suspected of having infectious endophthalmitis following cataract surgery and control subjects undergoing cataract procedures	128	73.5 (7–94)	NR	NR	Intraocular lens	POE risk factors and prevention	People with cataract surgery, posterior capsule tear, use of face masks, and subconjunctival antibiotics affect POE risk	Face masks and antibiotics are protective measures; posterior capsule tear increases risk
Khana et al., 2015	Observational	India	280	people with acute postoperative endophthalmitis following cataract surgery	NR	55.9 (10.9)	NR	NR	Phacoemulsification via scleral incision with foldable IOL	Risk factors for acute endophthalmitis	Posterior capsular rupture, ocular comorbidity, scleral incision with foldable IOL were risk factors	These were independent risk factors for acute endophthalmitis following cataract surgery
Sentamizh et al., 2022	Observational	India	60	People undergoing cataract surgery	32	64 (54–66.5)	NR	Culture positive (19), Culture negative (41)	extracapsular cataract extraction (ECCE), small-incision cataract surgery (SICS), and phacoemulsification	Visual outcomes after endophthalmitis	Factors like age, diabetes, absence of hypopyon, and microbiological culture results influence outcomes	Visual outcomes depend on clinical presentation and microbiological profile at the onset of endophthalmitis
Hou et al., 2023 [31]	Observational	Taiwan	1,263,497	People undergoing cataract surgery	499,260	62.02 (6.57)	NR	NR	Vitrectomy and intravitreal injection	Risk of POE in people with diabetes compared to people without diabetes	Diabetics had a slightly higher risk of POE (0.261% vs. 0.242%); dose–response relationship with DCSI > 10	Diabetes is a risk factor for POE; higher DCSI scores correlate with increased risk

### Outcomes

This study reviews synthesized data from multiple studies evaluating the incidence and risk factors of POE among people with and without diabetes undergoing cataract surgery. Silpa-Archa et al. (2019) [28] identified insulin therapy as the only significant risk factor for POE in people with diabetes, suggesting that further investigation into perioperative glycemic markers, such as preoperative glycated hemoglobin (HbA1c) and postoperative fasting plasma glucose levels, is warranted to better elucidate the role of glycemic control in POE risk. Similarly, Gondhale et al. (2021) [23] demonstrated that men with diabetes had 1.634 times higher odds of developing POE (P = 0.048), and diabetes combined with hypertension significantly increased the risk by 3.961 times (P < 0.001). The authors also reported an increased incidence of POE in people with diabetes who experienced a rapid reduction in their preoperative blood glucose levels, likely because patients with blood sugar levels > 200 mg required rapid reduction to meet the threshold for cataract surgery eligibility.

In a related context, Dev et al. (1999) [29] found that people with pre-existing diabetic retinopathy were at increased risk of disease progression and poorer visual outcomes following POE, supporting the hypothesis that inflammatory responses may exacerbate retinopathy in people with diabetes compared to those without diabetes. Hou et al. (2023) [31] further confirmed that people with diabetes exhibited a slightly higher incidence of POE than people without diabetes (0.261% vs. 0.242%, respectively), with the risk significantly increasing in those with a complication severity index (DCSI) score greater than 10.

Several studies have also identified risk factors for POE, irrespective of the diabetes status. For instance, Khanna et al. (2015) [27] and Sun et al. (2021) [24] highlighted that PCR significantly increased the risk of POE (OR, 6.98; 95% CI: 2.22–21.98). Additionally, ocular comorbidities and surgical factors such as phacoemulsification via scleral incision were found to increase the likelihood of POE.

Overall, people with diabetes, particularly those with coexisting hypertension, poor glycemic control, or advanced diabetic complications, face an elevated risk of developing POE. These findings emphasize the importance of comprehensive perioperative management, including meticulous glycemic control and consideration of systemic comorbidities, to mitigate the incidence of vision-threatening complications.

### Incidence rate of POE

**Fig. 2:**
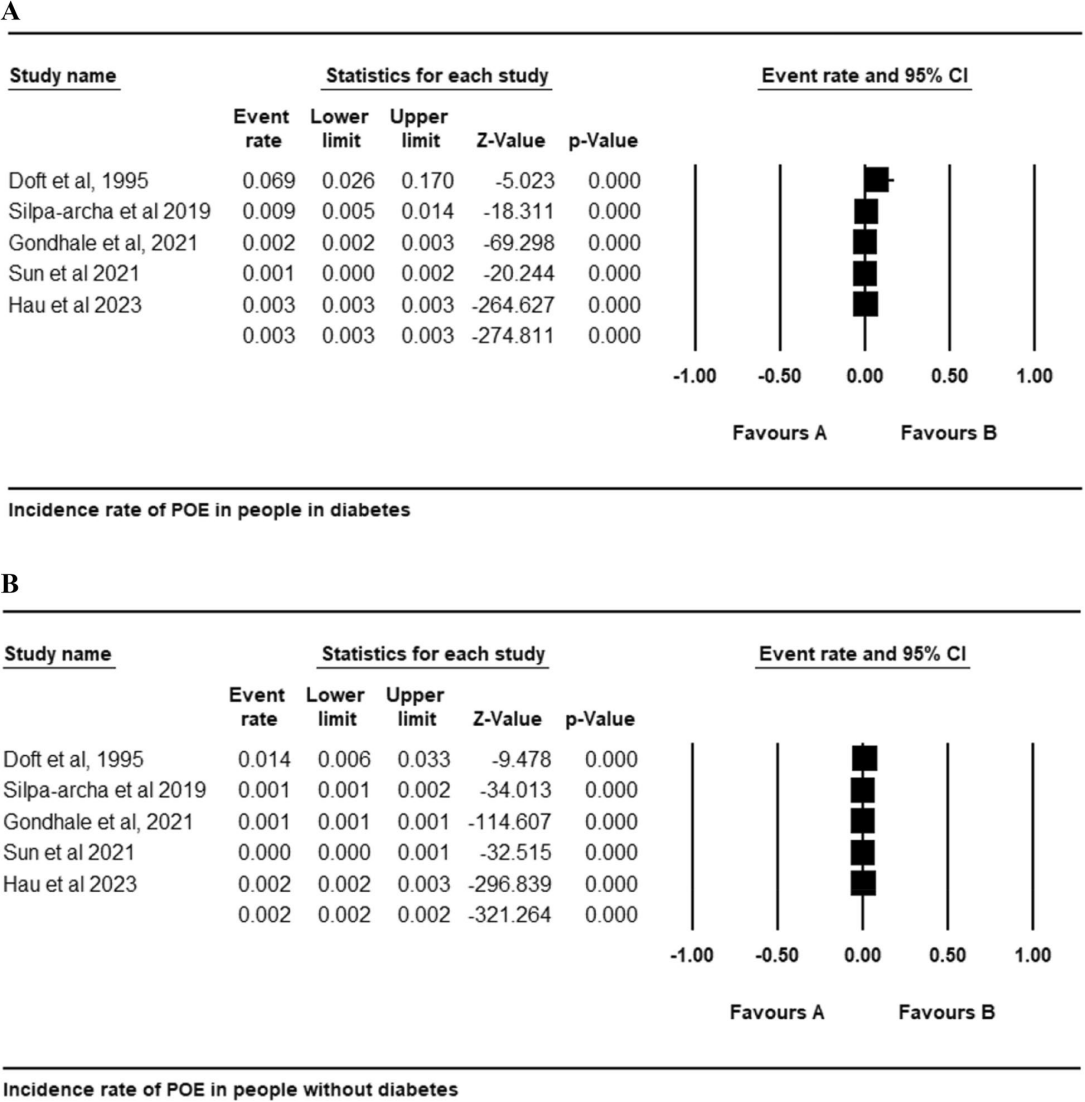
Incidence rate of POE in people with diabetes (**A**); Incidence rate of POE in people without diabetes (**B**)

**Fig. 3:**
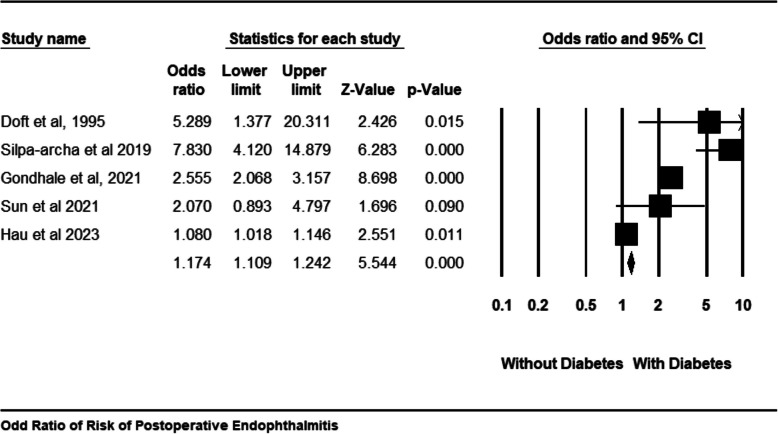
Odds Ratio of Risk of POE in people with diabetes versus people without diabetes

Forest plots in Figs. 2 and 3 indicate that patients with diabetes have a significantly increased incidence of POE following cataract surgery compared to those without diabetes. Specifically, Fig. 2A shows an incidence rate of 0.003 (95% CI: 0.003 to 0.003, p = 0.00) in people with diabetes, higher than the incidence rate of 0.002 (95% CI: 0.002 to 0.002, *p* = 0.00) observed in people without diabetes s, as illustrated in Fig. 2B. Furthermore, Fig. 3 highlights a statistically significant increase in risk, with a pooled odds ratio of 1.174 (95% CI: 1.109 to 1.242; p = 0.000), signifying that individuals with diabetes are 17.4% more likely to develop POE than those without diabetes. The absence of heterogeneity across studies (I^2^ = 0%) implies consistent findings, reinforcing that diabetes is an independent risk factor for POE after cataract surgery. These results emphasize the need for heightened preventive strategies in people with diabetes undergoing cataract procedures.

### Incidence rate of Posterior Capsule Rupture (PCR)

**Fig. 4:**
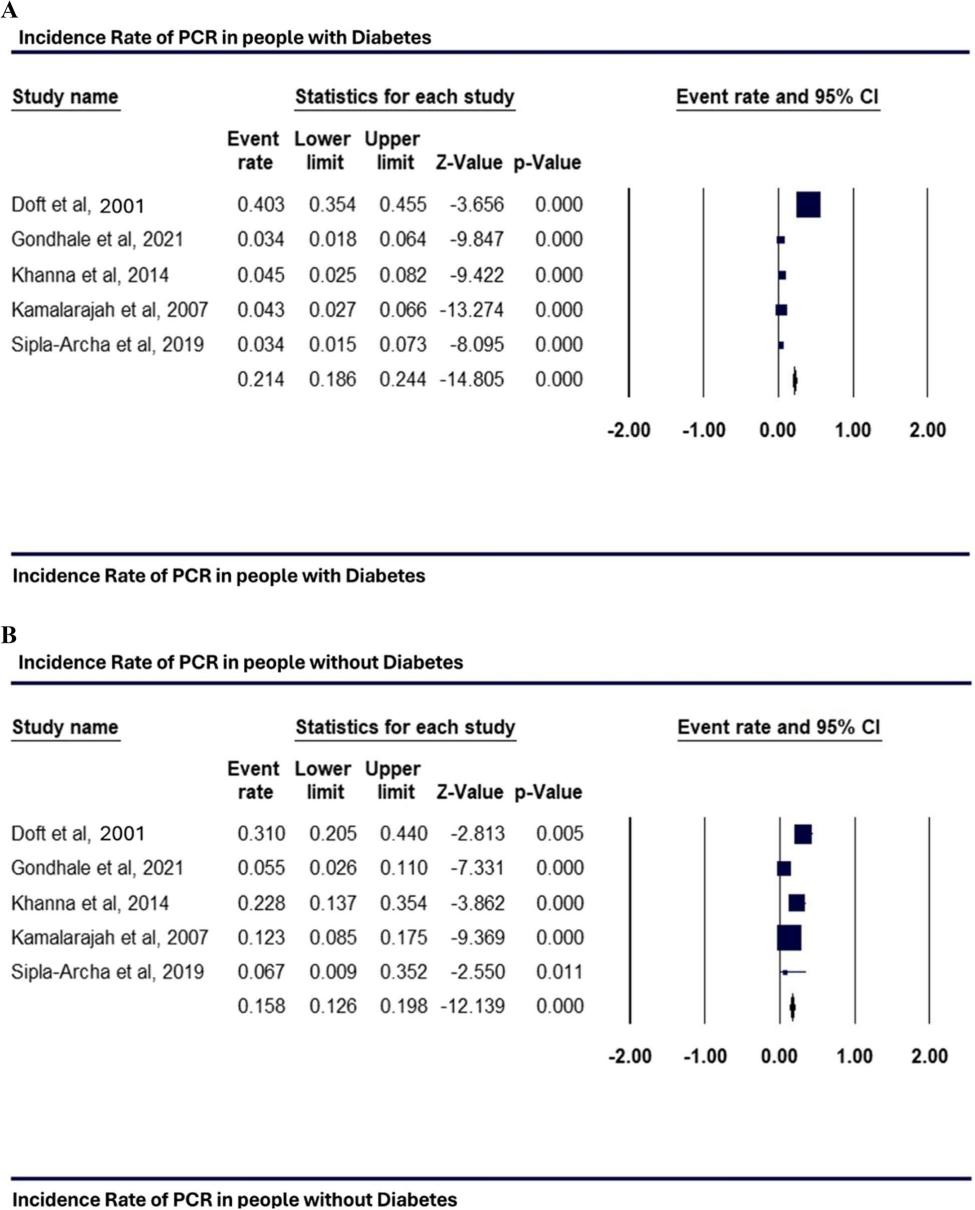
Incidence rate of PCR in people with diabetes (**A**); Incidence rate of PCR in people without diabetes (**B**)

**Fig. 5:**
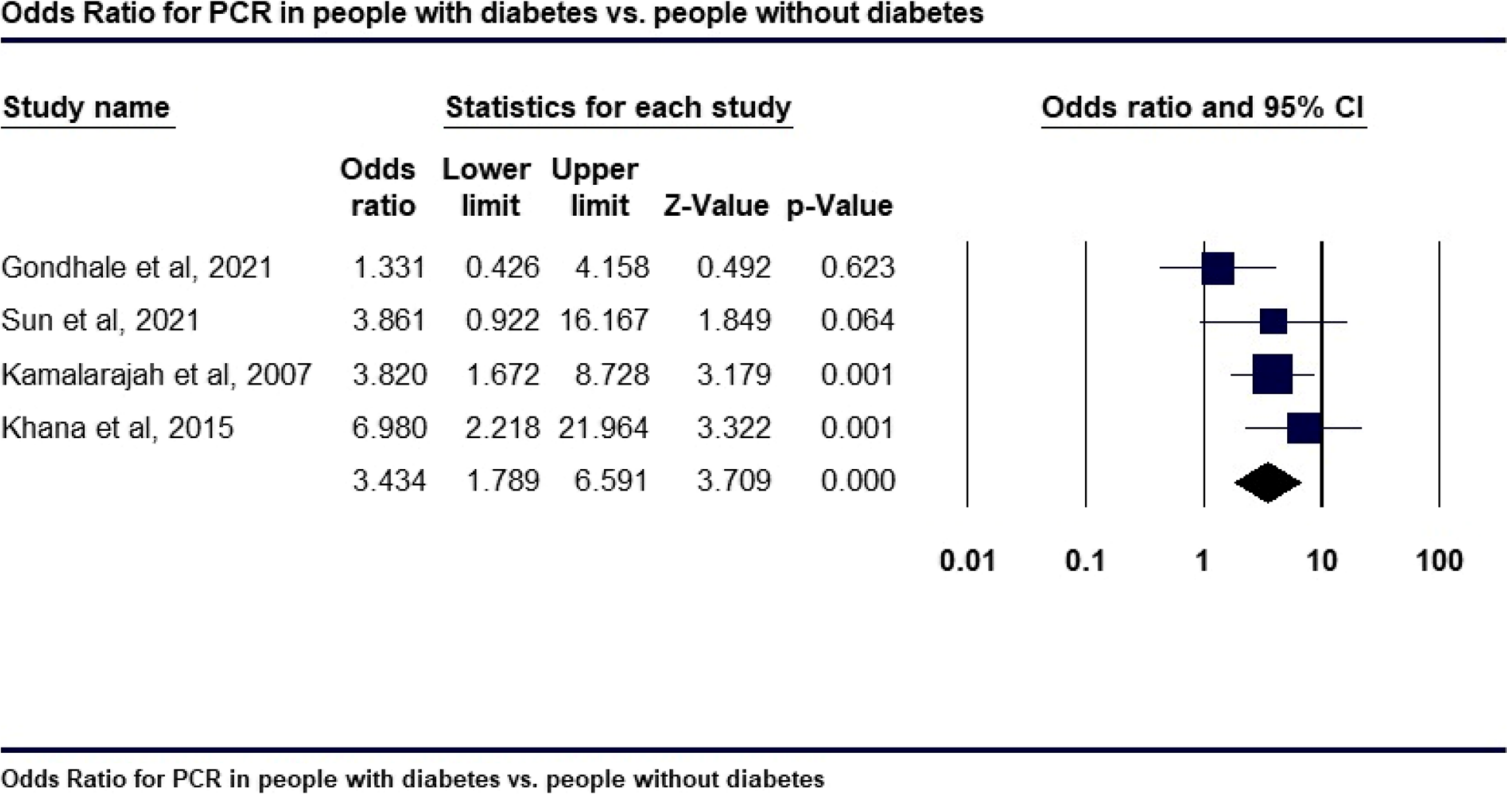
Odds Ratio for PCR in people with diabetes versus people without diabetes

Forest plots in Figs. 4 and 5 suggest that people with diabetes face a significantly higher risk of PCR during cataract surgery than those without diabetes. Figure 4A shows an incidence rate of 0.214 (95% CI: 0.186 to 0.244, p = 0.001) in people with diabetes, compared to a rate of 0.158 (95% CI: 0.126 to 0.198, p = 0.001) for those without diabetes, as shown in Fig. 4B. After adjusting for confounding factors, people with diabetes demonstrated a significantly increased likelihood of PCR, with a pooled odds ratio of 3.434 (95% CI: 1.789 to 6.591; p = 0.0001), indicating that they are over three times more likely to experience PCR than people without diabetes. The analysis showed moderate heterogeneity (I^2^ = 28%), suggesting some variability among study results, though the overall effect was statistically significant (Fig. 5). These findings highlight a greater vulnerability to PCR among people with diabetes during cataract procedures, underscoring the importance of cautious surgical approaches in this group.

### Efficacy of use of antibiotics on POE

**Fig. 6:**
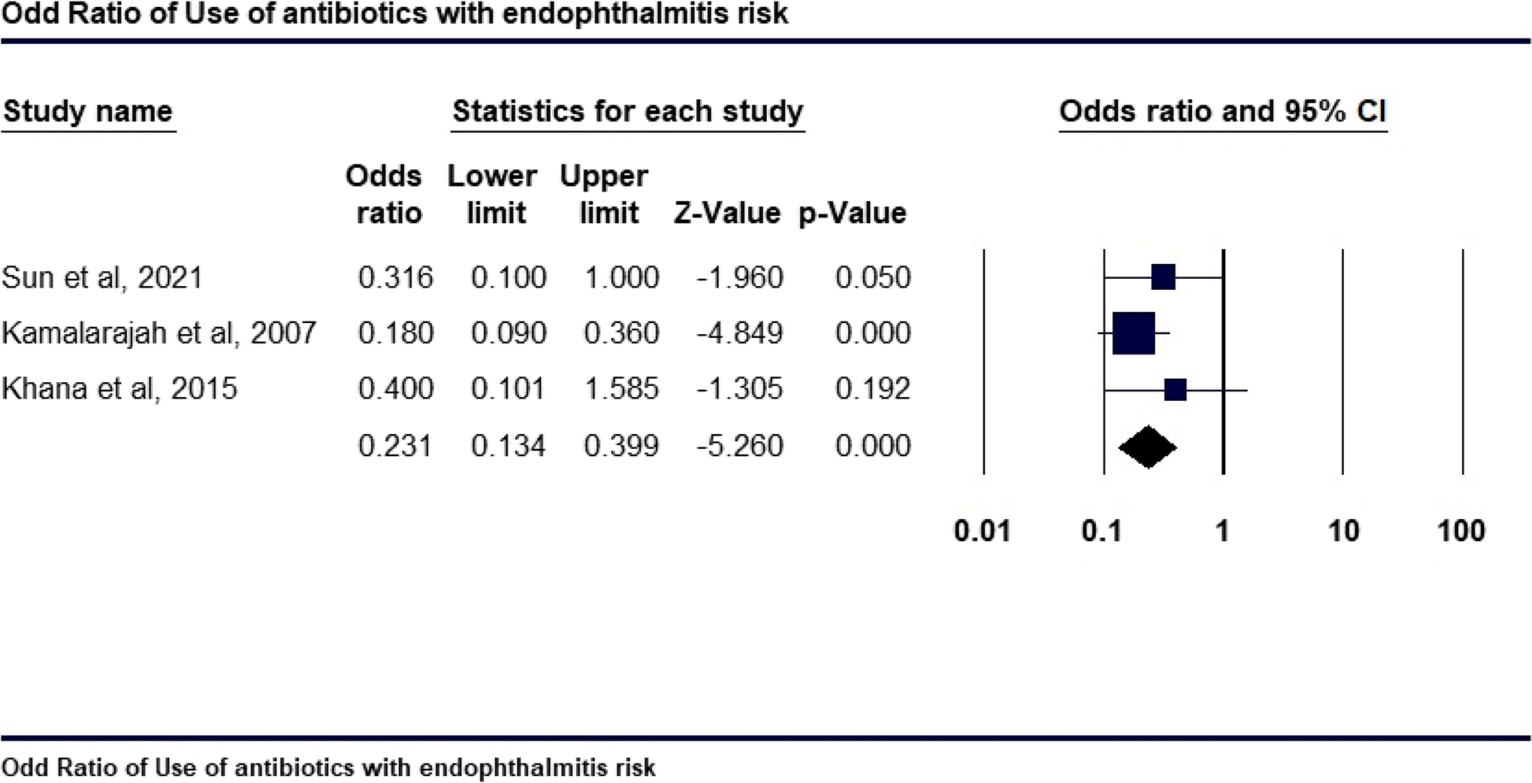
Odds Ratio of use of antibiotics with endophthalmitis risk in people with diabetes

Figure 6 presents a forest plot summarizing the association between antibiotic use and the risk of POE across three studies among people with diabetes. The studies by Sun et al. (2021), Kamalarajah et al. (2007), and Khana et al. (2015) each examine this relationship. The pooled analysis yields an OR of 0.231 (95% CI: 0.134–0.399, *p* = 0.000), demonstrating a significant protective effect of antibiotics in reducing the risk of POE by 23.1%. This finding supports the efficacy of postoperative antibiotic use in lowering this risk, with most studies showing statistically significant results. Specifically, the use of intracameral and topical antibiotics, such as vancomycin, cefazolin, and moxifloxacin, has been shown to significantly reduce the risk of POE in both people with and without diabetes (OR: 0.231; p = 0.00). A network meta-analysis by Kato et al. (2022) [32] concluded that the use of antibiotics after cataract surgery significantly reduces the risk of POE compared to no antibiotic prophylaxis. Intracameral vancomycin demonstrated the greatest reduction in risk, with a 97% decrease (OR 0.03), followed by cefazoline (91% reduction, OR 0.09), cefuroxime (82% reduction, OR 0.18), and moxifloxacin (64% reduction, OR 0.36). In comparison, people with diabetes receiving postoperative antibiotic prophylaxis, especially vancomycin, experienced a substantial reduction in the incidence of POE, though their outcomes were still less favorable than those of people without diabetes who received the same prophylactic treatment.

### Risk of bias

Quality assessment of the included studies was conducted using the Risk of Bias in Non-randomized Studies of Interventions (ROBINS-I) tool and the Cochrane risk-of-bias tool for randomized trials (RoB 2.0). The findings indicate varying levels of risk across studies (Table 2).

**Table 2:** Quality assessment of included studies in systematic review and meta-analysis

Risk Of Bias in Non-randomized Studies of Interventions (ROBINS-I)								
	**Bias due to confounding**	**Bias in selection of participants into the study**	**Bias in classification of interventions**	**Bias due to deviations from intended interventions**	**Bias due to missing data**	**Bias in measurement of outcomes**	**Bias in selection of the reported result**	**Overall bias**
**Silpa-archa et al., 2019 **[28]	L	M	M	N	M	L	M	M
**Gondhale et al., 2021 **[23]	L	L	L	L	L	L	L	L
**Sun et al., 2021 **[24]	L	M	L	L	M	L	L	L
**Dev et al., 1999 **[29]	L	S	S	M	L	L	M	S
**Kamalarajah et al., 2007 **[26]	L	L	N	M	L	L	M	M
**Khana et al., 2015**	L	L	L	L	L	L	L	L
**Sentamizh et al., 2022**	L	S	S	M	L	L	L	S
**Hou et al., 2023 **[31]	L	L	L	L	L	L	L	L
**Version 2 of the Cochrane risk-of-bias tool for randomized trials (RoB 2.0)**								
	**Bias arising from the randomization process**		**Bias due to deviations from the intended interventions**	**Bias due to missing outcome data**	**Bias in measurement of the outcome**		**Bias in selection of the reported result**	**Overall bias**
**Doft et al., 2001 **[25]	+		** + **	+	**O**		**O**	** + **

For ROBINS-I: *N* no information, *L* low risk of bias, *M* moderate risk of bias, *S* serious risk of bias, *C* critical risk of bias

For RoB 2.0: + , low risk of bias; O, some concerns; − , high risk of bias

For the ROBINS-I assessment, most studies demonstrated a low risk of bias, particularly those by Gondhale et al. (2021) [23], Khanna et al. (2015) [27], and Hou et al. (2023) [31] consistently scored low across all the domains. In contrast, Silpa-Archa et al. (2019) [28], Sun et al. (2021) [24], and Senthamizh et al. (2022) [30] presented moderate risks in certain areas, such as participant selection and the classification of interventions, which may introduce potential biases. Notably, Dev et al. (1999) [29] and Kamalarajah et al. (2007) [26] reported serious risks in the selection of participants and classification of interventions, indicating that these factors should be considered when interpreting their results.

The RoB 2.0 assessment for randomized trials revealed that Doft et al. (2001) [25] exhibited low risks in several domains, although some concerns were noted regarding deviations from the intended interventions and missing outcome data. Overall, the varying levels of bias across studies underline the necessity for cautious interpretation of the findings, particularly regarding studies with higher risks of bias.

### Publication bias

**Fig. 7:**
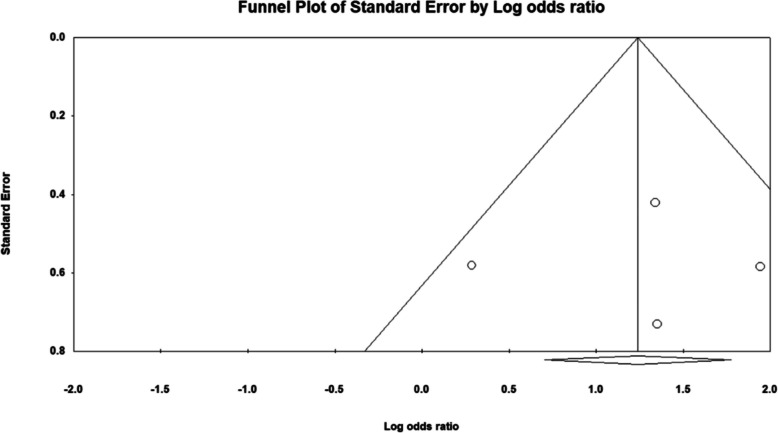
Funnel plot for posterior capsule rupture

**Fig. 8:**
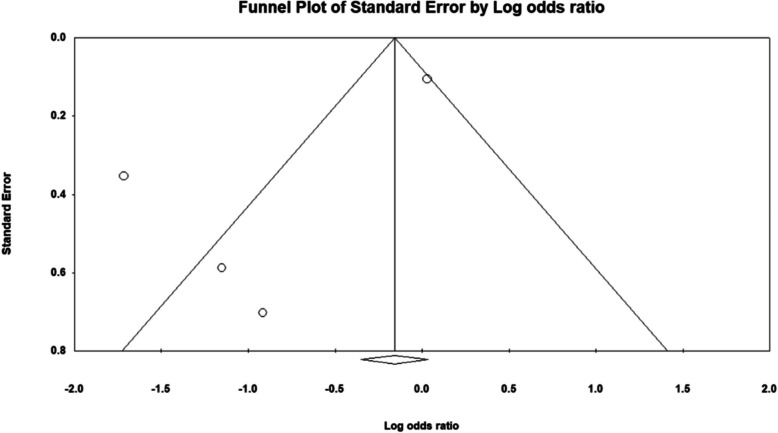
Funnel plot for antibiotic use and endophthalmitis risk

Publication bias was assessed using funnel plots and Egger's test for both the outcomes. For the analysis of posterior capsule rupture, the funnel plot displayed slight asymmetry, suggesting potential publication bias, with an Egger intercept value of −0.32 and a p-value of 0.46, indicating that the bias was not statistically significant. (Fig. 7) Similarly, the evaluation of antibiotic use and its association with endophthalmitis risk also showed slight asymmetry in the funnel plot, with an Egger intercept value of −3.2 and a p-value of 0.09. (Fig. 8) While these findings indicate some degree of asymmetry, the non-significant p-values suggest that publication bias may not substantially affect the overall results of this systematic review. Nevertheless, the observed asymmetries warrant caution in the interpretation of these outcomes, and further investigation of the factors contributing to this asymmetry may be beneficial.

## Discussion

To the best of our knowledge, this is the first meta-analysis aimed to improve perioperative care strategies and patient outcomes by recognizing modifiable risk factors and enhancing treatment protocols for people with diabetes undergoing cataract surgery. This systematic review and meta-analysis primarily aimed to assess the incidence and risk factors of POE in people with diabetes versus people without diabetes undergoing cataract surgery. A thorough review of the literature yielded nine studies meeting the inclusion criteria, with seven selected for meta-analysis. The studies exhibited considerable diversity in their design, participant demographics, and methodological approaches; however, all focused on outcomes related to cataract surgery, particularly in people with diabetes. Individuals with diabetes, especially those with advanced diabetic complications, were found to have an increased likelihood of developing POE compared with people without diabetes. Additionally, the risk of PCR during cataract surgery is notably higher in people with diabetes. Although antibiotic use generally provided protection, no statistically significant difference was observed in effectiveness between the people with diabetes and people without diabetes groups. These results highlight the need for meticulous perioperative management, including the control of systemic factors such as blood glucose levels and hypertension, to reduce the risk of endophthalmitis in people with diabetes.

The incidence of POE has been reported in our study as significantly higher in people with diabetes compared to those without diabetes. This trend, observed in our analysis, aligns with previous findings suggesting that systemic conditions associated with diabetes may predispose individuals to POE [33]. While the pathophysiological mechanisms underlying this increased susceptibility remain unclear, it is hypothesized that people with diabetes’ altered immune response and impaired wound healing may contribute to the elevated risk [34]. Our findings highlight the need for tailored risk assessment in people with diabetes undergoing cataract surgery to minimize the likelihood of postoperative infections and improve outcomes.

PCR is a known risk factor for POE and is observed at a higher rate in people with diabetes. Our analysis indicates that the incidence of PCR is significantly elevated in people with diabetes compared to people without diabetes. The increased PCR risk in people with diabetes may be attributed to factors such as altered lens anatomy and the greater prevalence of comorbidities that complicate surgery [35]. Consequently, PCR in people with diabetes may amplify the risk of POE, as the rupture creates a pathway for microbial entry, heightening the infection risk [36]. These findings underscore the importance of meticulous surgical technique and preoperative assessment in people with diabetes to reduce PCR-related POE risk. Diabetes mellitus impairs neutrophil chemotaxis and phagocytosis, essential processes for protecting against bacterial invasion during cataract surgery. This impairment leads to a slower and weaker response to contamination [37]. Additionally, abnormalities in the complement system among people with diabetes further weaken their immune defense, raising the risk of bacterial growth and infection [38]. Hyperglycemia negatively affects wound healing by impairing collagen synthesis and fibroblast function, potentially allowing prolonged exposure to pathogens [39]. Hyperglycemia-induced changes in blood vessel function further impair tissue repair and increase the risk of postoperative complications including endophthalmitis. Excess glucose in tissues and fluids enhances bacterial proliferation, particularly during cataract surgery, where minor contamination can occur [40].

People with diabetes are more susceptible to POE, with Staphylococcus epidermidis, Staphylococcus aureus, and Streptococcus species being common causative organisms [41]. Hyperglycemia may promote biofilm formation in surgical instruments or intraocular lenses, contributing to persistent infections and treatment resistance [42]. Coagulase-negative staphylococci (CNS), particularly S. epidermidis, are the main pathogens in POE [43]. Altered immune function, impaired wound healing, and increased exposure to resistant bacteria in hospital settings elevate infection risk, especially in people with diabetes with frequent hospitalizations or antimicrobial use [44]. In our meta-analysis, the protective effect of antibiotics on POE risk appears consistent across people with diabetes and people without diabetes, though the impact shows variability depending on antibiotic type, route, and timing [45]. However, the incidence rate of POE among people with diabetes, with and without the use of antibiotics, is not fully reported in studies included in the meta-analysis. Future research should focus on standardized antibiotic regimens for high-risk populations to better understand the protective role of antibiotics and potentially enhance POE prevention in people with diabetes. Antibiotic prophylaxis often includes vancomycin, cefazolin, and moxifloxacin. Vancomycin is effective against methicillin-resistant staphylococci [46], while cefazolin targets gram-positive bacteria [47]. However, moxifloxacin’s effectiveness is limited against CNS, and resistance is rising, especially in diabetes [48]. Further research is needed to refine prophylaxis strategies.

People with diabetes frequently suffer from microvascular complications such as diabetic retinopathy, which compromises blood flow to ocular tissues [49]. Advanced diabetes causes tissue ischemia, reduces oxygen supply to surgical sites, and impairs immune function and tissue repair. Ischemic ocular tissues struggle to fight infections and heal, increasing the risk of POE after cataract surgery. Research shows that people with diabetic retinopathy are more likely to have poor visual outcomes post-endophthalmitis due to pre-existing retinal damage [50]. Hyperglycemia in diabetes disrupts the blood-aqueous barrier, increasing vascular permeability, inflammation, and susceptibility to eye infections like endophthalmitis. In diabetes, increased sorbitol accumulation accelerates cataract formation and may worsen post-operative eye outcomes [51]. Our study supports the conclusions drawn by Shi et al. (2022) [52], who reported a greater occurrence of POE in people with more severe cataracts. This connection underscores the necessity for developing tailored surgical approaches and implementing more stringent post-operative surveillance for people with diabetes, particularly those with advanced cataracts.

This research has several strengths, including a thorough examination of the literature across various databases, ensuring a comprehensive selection of studies. By concentrating on people with diabetes, a group at elevated risk, this investigation addresses a crucial clinical issue regarding post-surgery complications. This study incorporated both qualitative and quantitative analyses, offering an in-depth examination of the occurrence and risk factors associated with POE. The research also emphasizes the link between PCR and POE, underscoring the importance of precise surgical methods to reduce complications in people with diabetes.

This research encounters several limitations. Variations in study designs, participant profiles, and methodologies may introduce inconsistencies, limiting the broader applicability of the results. The meta-analysis's statistical robustness and reliability were restricted by the variation in sample size of seven studies in the quantitative evaluation. The reliance on observational data instead of randomized controlled trials introduces potential biases that may compromise the findings' validity. Additionally, inconsistencies in reporting diabetes status, including type and control levels, among included participants may affect POE risk. Uniform documentation of diabetes severity and duration was lacking across studies, despite the known association between prolonged diabetes duration, poor blood glucose control, and increased postoperative infection risk. Inconsistent accounting for medications used by people with diabetes, including insulin and other glucose-lowering agents, limits the assessment of their impact on POE risk. The presence of comorbidities like hypertension and cardiovascular disease, which frequently accompany diabetes, may confound the association with POE.

## Conclusion

Our meta-analysis shows that people with diabetes have a significantly higher incidence of POE and PCR following cataract surgery compared to those without diabetes. Specifically, the odds ratio for POE is 1.174, indicating a 17.4% increased risk in people with diabetes. For PCR, the incidence rate is 0.214 for people with diabetes versus 0.158 for those without, with an odds ratio of 3.434, reflecting more than a threefold increased risk of PCR in people with diabetes compared to those without diabetes. Antibiotic use has a significant protective effect for both groups, reducing the incidence of POE in those receiving prophylaxis. Our study indicates that for people with diabetes undergoing cataract surgery, it is crucial to maintain well-controlled blood sugar and blood pressure beforehand. During surgery, extra caution should be taken to prevent PCR, and appropriate antibiotic use should be considered to minimize the risk of POE.

## Data Availability

No datasets were generated or analysed during the current study.
